# Ballistic impacts on an anatomically correct synthetic skull with a surrogate skin/soft tissue layer

**DOI:** 10.1007/s00414-017-1737-9

**Published:** 2017-11-28

**Authors:** Peter Mahoney, Debra Carr, Richard Arm, Iain Gibb, Nicholas Hunt, Russ J. Delaney

**Affiliations:** 1 Royal Centre for Defence Medicine, ICT Centre, Research Park, Birmingham, B15 2SQ UK; 2 Centre for Defence Engineering, Cranfield University at the Defence Academy of the United Kingdom, Shrivenham, Swindon, SN6 8LA UK; 3 Flexural Composites Research Laboratory, 107 Bonington Building, Nottingham Trent University, Dryden Street, Nottingham, NG1 4GG UK; 4 X-ray Department, Medical Centre, HMS Nelson, HM Naval Base Portsmouth, Hampshire, PO1 3HH UK; 5 Forensic Pathology Services, Grove Technology Park, Wantage, Oxon OX12 9FA UK; 6 South West Forensic Pathology Group Practice, Box 388, Bristol, PO BS9 0DB UK

**Keywords:** Head injury, CT scanning, Ballistic images, Synthetic skin

## Abstract

The aim of this work was to further develop a synthetic model of ballistic head injury by the addition of skin and soft tissue layers to an anatomically correct polyurethane skull filled with gelatine 10% by mass. Six head models were impacted with 7.62 x 39 mm full metal jacket mild steel core (FMJ MSC) bullets with a mean velocity of 652 m/s. The impact events were filmed with high-speed cameras. The models were imaged pre- and post-impact using computed tomography. The models were assessed post impact by two experienced Home Office pathologists and the images assessed by an experienced military radiologist. The findings were scored against real injuries. The entry wounds, exit wounds and fracture patterns were scored positively, but the synthetic skin and soft tissue layer was felt to be too extendable. Further work is ongoing to address this.

## Introduction

Ballistic head injury is a significant threat to troops in combat [1] and ongoing research is needed to assist designers of military helmets and associated personal protective equipment [2].

The Impact and Armour Group at Cranfield University, Defence Academy of the UK are working on an anatomically correct synthetic model of ballistic head injury for this purpose. Preliminary work has been reported [3] along with a further development assessing the fracture patterns produced in the model under ballistic impact for clinical realism [4].

An acknowledged limitation of the model to date has been the lack of skin and soft tissue layers around the synthetic skull.

Thali et al. [5] developed a ‘skin-skull-brain model’ made of a silicone scalp, a layered polyurethane sphere to represent the skull, and gelatine 10% at 4 °C to simulate brain. After shooting the model with a series of ammunition types (9 mm Luger Full Metal Jacket, FMJ, 22LR, .38Spl, .44 Rem Mag, 7.62 × 51 mm NATO FMJ, 7.62 x 39 mm FMJ and 12/70 Brenneke Slug), the authors reported that the results were comparable to those of real gunshot injuries.

### Gunshot wound characteristics

The appearance and characteristics of gunshot wounds depend on a number of factors. These include (i) weapon type, (ii) projectile type, (iii) projectile velocity, (iv) distance of the weapon from the person when fired, (v) the effect of intermediate targets such as clothing or armour, and (vi) where on the body the person was struck. Bullets impacting on soft areas such as muscle may produce different appearances to those impacting hard areas (e.g. where bone is close to the body’s surface such as the head). This is explored in standard forensic textbooks, e.g. [6].

**Fig. 1:**
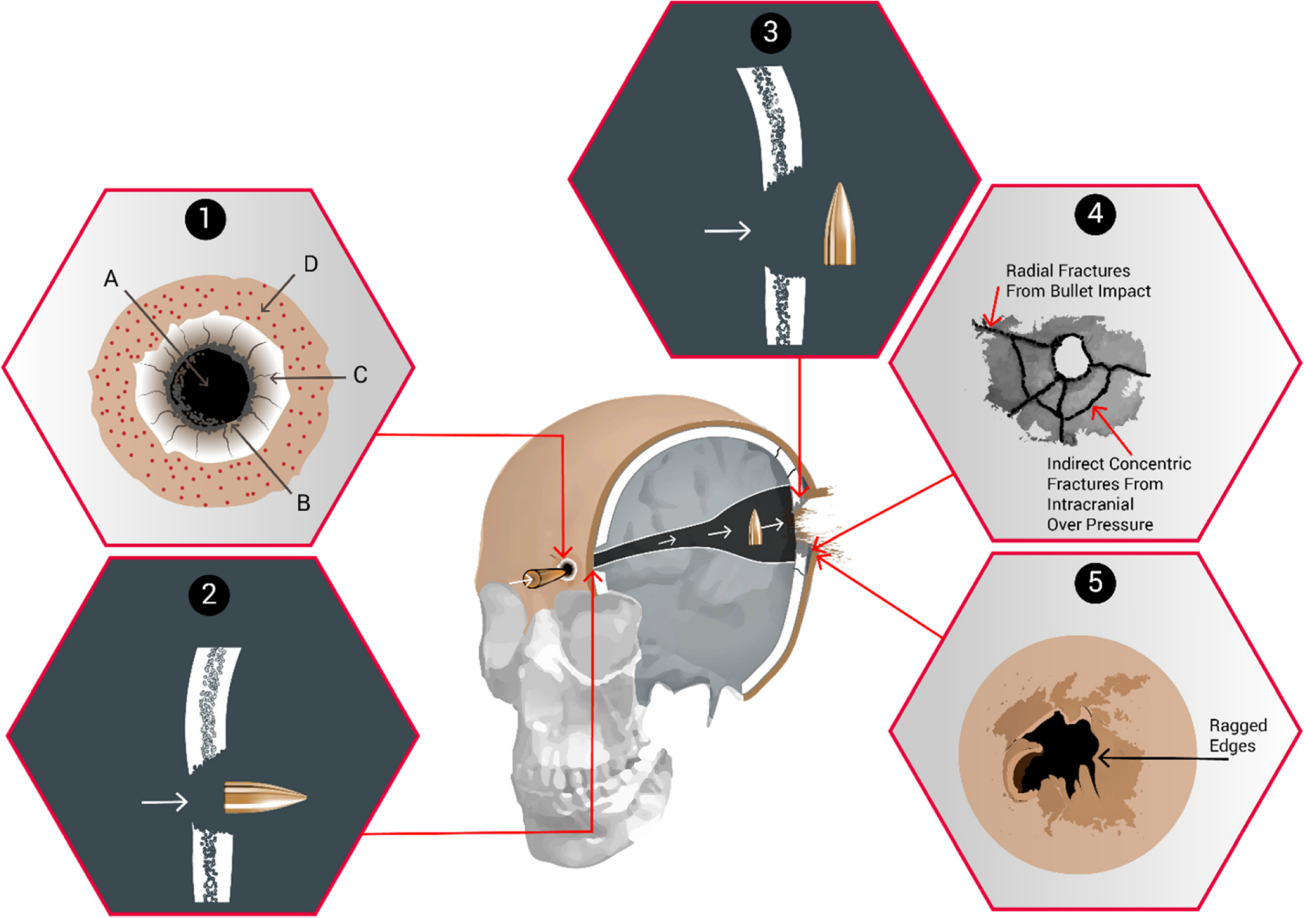
Head injury gunshot characteristics. 1. Skin and soft tissue entry wound, after Thali et al. [7]. For explanation of letters A-D please see text. 2. Bone entry wound, internal bevelling. 3. Bone exit wound, external bevelling, (2 and 3 after DiMaio [8]). 4. Additional bone fractures (after Karger [9]). 5. Skin and soft tissue exit wound

Thali et al. [7] used their model to look at the characteristics of (non-contact) gunshot entrance wounds produced by 9-mm Luger FMJ fired 10 m from the target with a muzzle velocity of 350 m/s. These characteristics are summarised in Fig. 1. Thali et al. [7] noted that the terminology is not uniform among different authors and Fig. 1 is an attempt to reconcile this.

Explanation of Fig. 1:

1. Skin and soft tissue gunshot entry wound (after Thali et al. [7])
  A. Central ‘defect’ due to (i) tissue destruction by the bullet and (ii) tissue compression as the skin is spread radially by the impact.
  B. ‘Bullet wipe’, a ring of contamination, due to materials on the head of the projectile (e.g. dirt, oil, propellant) being transferred to the skin (Bullet wipe may also be found on the underlying bone)
  C. Abrasion collar/Contusion ring [6, p258], which has different mechanisms proposed for its creation [6, 7, 10]. Thali proposes it is due to temporary over extension of the skin adjacent to the impact area, and the skin subsequently drying out. Rothschild [11, p258] describes radial stretching cracks and tears in the epidermis.
  D. Margin of distension. Rothschild [11, p258] refers to this as being the boundary of the skin stretched by the radial acceleration forces and is associated with petechial haemorrhage.
2. Entrance wound bone damage (After DiMaio [8])
  The bone of the cranial vault is made up of outer and inner cortical tables joined by thin cancellous bone (the Diploë) [12, p673]. The ‘typical’ appearance of an entry wound is that of a ‘broadening cone’ [12, p674] or crater [6, p261]. This is described as ‘internal beveling’ [8]. In a review of the skeletal remains of 21 gunshot victims, Quatrehomme and İșcan [13] found internal beveling in the bone entry wounds of 20 skulls but noted external beveling in one.
3. Exit wound bone damage (after DiMaio [8])
  If the bullet has sufficient energy to cross the skull and perforate bone again, a similar action occurs but with the broader aspect of the wound on the outside of the skull (‘external bevelling’) [12, p674]. In this case, the bullet has ‘yawed’ within the brain tissue and exited side on causing the bone exit wound to be larger than the entrance. Quatrehomme and İșcan [13] noted bone exit wounds to be more irregular than bone entry wounds and found external beveling in most vault injuries but not those of the orbit, maxilla, temporal, greater wing of the sphenoid or left occipital bone.
4. Additional fractures (after Karger [9])
  The bony injury seen may be complicated by further fractures. Karger [9, p151] describes how secondary radial fractures are induced by the bullet’s impact and originate at the entry and exit sites. Karger also describes how the brain is vulnerable to cavitation [9, p149] but the intact skull does not allow expansion, resulting in high pressures within the cranial cavity. If the overpressure exceeds the skull’s capacity to elastically extend, indirect concentric fractures result. Sufficiently high pressures will result in fractures combining to produce an ‘explosive’ type of injury [6, 9, 11, 14].
5. Skin and soft tissue exit wounds
  Rothschild [11, p260] notes that exit wounds show a high degree of variation. In a perforating injury, the skin bulges out before breaking producing an irregular, slit-like or stellate wound with everted edges. Deformed bullets and fragmented bullets are associated with more skin tearing [11, p261]. The exit wound associated with a high energy round producing a temporary cavity will vary with the length of the wound tract and whether the exit point occurs within or after the temporary cavity [6, p262; 11, p261].

### Imaging in ballistic investigations

Thali et al. also described using their model to look at fracture pattern development from a 9-mm bullet impact [15]*.* The impact sequence was captured with high-speed photography and the model underwent radiographic computed tomography (CT) examination to visualise the wound tracts and fractures. The images were, in turn, compared to the findings when the model underwent ‘autopsy’. The authors concluded that the model produced realistic features of gunshot injury and that the CT examination and the ‘autopsy’ revealed very similar data. They also postulated the role of imaging for ‘virtual’ autopsies [15].

Imaging studies have gone hand in hand with experiments to understand ballistic injury mechanisms.

Butler et al. [14] describe using an X-ray apparatus with an exposure time of 1 microsecond to capture temporary cavity formation in the brains of anaesthetised animals (cats and dogs) impacted by steel spheres at between 3800 and 4000 ft/s.

Watkins et al. [16] used a model consisting of dried human skulls filled with 20% gelatine and covered with two layers of gelatin soaked chamois leather. The models were impacted with either 3 or 6 mm diameter ball bearings (with velocities between ~200 to 1300 m/s, [16, p S43, Table III]) in a series of 12 experiments. In the later experiments, a pulsed X-ray source was used to produce a train of 50 images at millisecond intervals during the impact events and a cine camera used to capture the resulting images.

Other authors have used CT imaging for ballistic experiments. Schyma et al. [17] constructed four head models using hollow spheres filled with 10% gelatine. Thin foil bags containing a mixture of acryl paint and barium meal were glued onto each sphere and the assembly coated with a layer of silicone. The models were shot through the foil bag with 9 x 19 mm pistol ammunition and the following day underwent CT examination. The barium within the wound tract allowed reconstructed 3-D images of the damage to be created. They also removed the gelatine cores from the models after shooting, cut them into 1 cm slices and scanned the slices on a flatbed scanner to produce scanned images of the bullet tract. They found the correlation of the optical and radiological measurements to be ‘satisfactory’ [17].

Bollinger et al. [18] used CT imaging to assess damage in a SYNBONE® pelvis embedded in 10% gelatine and impacted with 9 mm and .45-in. pistol ammunition. The authors felt that CT imaging offered advantages over dissecting the model as (i) the distribution of osseous fragments within the gelatine could be observed more accurately and (ii) the crack lengths within the damaged gelatine could be measured allowing assessment of energy transfer along the bullet’s course.

Karger et al. [19] had a licenced veterinarian shoot 10 live New Jersey calves (destined for the slaughter house) with either 9 x 19 mm FMJ or 9 x 19 mm hollow point ammunition in the right temple. The heads underwent full autopsy and the brains were removed and fixed in formaldehyde. The fixed brains were imaged using plain X-ray, CT and magnetic resonance imaging (MRI). Each brain was also examined and histology performed. Key features of the brain injury included wound tracts due to direct tissue crushing by the bullet, cortical contusions from the brain impacting against the inside of the skull, shearing of brain tissue from the intracranial temporary cavitation, associated oedema and bruising, and bone fragments both within the wound tracts and driven into brain tissue.

Oehmichen et al. [20] studied 47 cases of lethal gunshot injury to the brain from civilian practice. In 17 of these, CT and MRI were performed either prior to autopsy or on the isolated formalin fixed brain, and the imaging correlated with the autopsy findings [21]. They reported that imaging was able to distinguish entrance from exit wounds, determine the missile track and demonstrate aspects of the brain injury. CT was particularly useful in localising foreign objects within the brain (e.g. bone and bullet fragments) which can be difficult to locate during autopsy [21].

### This project

The aim of this current work was to assess the effect of synthetic facial skin and tissue on the fracture development in our model and assess the overall realism of the entry wounds, exit wounds, wound tract, fractures and tissue characteristics using both modern CT scanning and formal ‘autopsy’, building on the approach of Thali and colleagues. Assessment of entry and exit wound characteristics was felt to be a key observation given the reported variation of wound appearances in both forensic [22] and experimental [23] cases.

## Methods

The research described in this paper was carried out in a number of stages.

i. Skulls

Six anatomically correct polymeric skulls were manufactured from rapid prototype data obtained by 3-D mapping of both the internal and external surfaces of a human skull (ARRK Europe Ltd., Gloucester Technical Centre, Olympus Park, Quedgeley, Gloucester, Gloucestershire GL2 4NF).

Synthetic bone surrogates have been assessed by other authors for ballistic testing [24, 25] and synthetic skulls produced realistic fracture patterns in our previous work [3, 4].

The polymer used for this work, MU51, has been previously described [4]. The skulls are made from a two-part thermoset polyurethane plastic mixed together in the correct ratios within a vacuum casting chamber (Craig Vickers, ARRK Europe Ltd., personal communication). The skulls are produced in two parts (above and below the post-mortem cut line) and need to be bonded prior to ballistic tests. The glue line was noted to be a weak point in previous work [4] so a number of adhesives were assessed for suitability under ballistic strain conditions. Pro-Flex 50, a 50 Shore A fast curing rubber (http://www.mouldlife.net/ekmps/shops/mouldlife/resources/Other/pro-flex-50-data-sheet.pdf), has, to date, proved the most effective. The two parts of the skulls were bonded at the Flexural Composites Research Laboratory, Nottingham Trent University (FCRLNTU).

ii. Faces

Sheets of polydimethylsiloxane (PDMS) composite prepared as a surrogate skin/subcutaneous tissue were produced by FCRLNTU for a previous set of ballistic experiments [26]. Samples of the surrogate skin/subcutaneous tissue were assessed using the BS ISO3–1:2015 Trouser Tear test and by measuring Shore hardness. The Shore hardness was similar to reported values [27] for human skin, pig skin and dental silicones but tear strength was lower [26].

The PDMS surrogate skin/subcutaneous tissue was derived from part of a larger work strand to build realistic artificial skin and organs to support military surgical training [28]. This project involved creating surrogate samples to mimic the tactile qualities of real living tissues such as muscle, liver and lung. Surgeons and other clinicians were invited to comment on how ‘real’ particular synthetic tissues appeared to them and the synthetic materials adjusted accordingly [29]. Previous work within the Impact and Armour Group has used food-grade swine tissue for ballistic experiments [30] and assessing the PDMS against this was felt to be a useful comparison.

When impacted by the same Ukrainian 7.62 × 39 mm Mild Steel Core (MSC) rounds as selected for the current experiment, a combination of the PDMS surrogate with sheets of MU51 polymer produced very similar results to horse scapulae with a residual layer of tissue [26]. The same combination of materials was therefore chosen for this experiment as a suitable skin/soft tissue/polymer bone substitute, while accepting that the polymer skull lacks the complex structure of real bone [24].

**Fig. 2:**
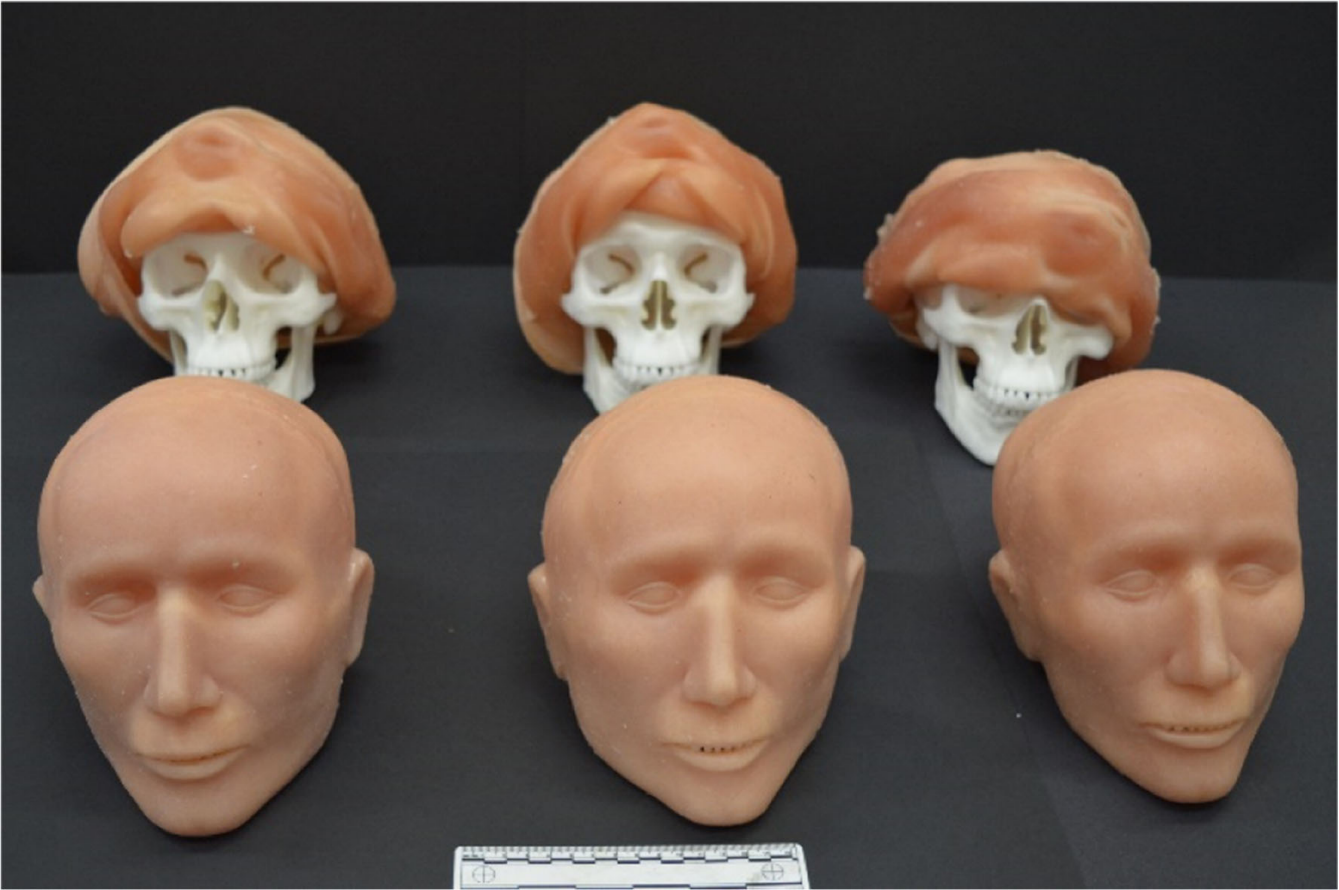
MU51 polymer skulls with moulded PDMS faces and scalps

Using anatomical data [31], the facial tissues were built up layer by layer on one of the synthetic skulls using a wax-based polymer clay. The final model was the base from which moulds were created for the PDMS tissue structures used to form faces and scalps to place over the skulls [32] (Fig. 2).

iii. Complete model

A thin low-density polyethylene bag was inserted into the base of the face/skull model and gelatine, 10% by mass, poured into the bag to fill the cranial cavity. The gelatine was allowed to set for 24 h at around 17 °C. Our previous work did not find a difference in the fracture patterns generated in a skull model filled with gelatine 10% at a series of temperatures [4] and therefore no further temperature conditioning was used. While accepting that 10% gelatine is not a completely biofidelic brain stimulant [33], its use for the current project allows reference to our previous work [4, 26]. Jussila [34] notes that the properties of tissue simulants do not need to be exactly the same as living tissue ‘provided the results can be measured and appropriately extrapolated or scaled’ [34].

iv. First set of CT scans

The complete face/skull models with gelatine fill were taken to the Centre for Defence Imaging (Royal Centre for Defence Medicine, Queen Elizabeth Hospital, Birmingham) and underwent CT scans (SOMATOM Definition CT scanner, Siemens Health Care Ltd., Camberley, UK) using both Dual Energy Head Angiogram and Spiral Head protocols (Window Level 100/35, 1-mm slice thickness). The models were given designations using the NATO Phonetic Alphabet [35] Golf (Face 1) through to Lima (Face 6) to allow images to be catalogued and filed.

The CT scans produced pre-impact images for each model, allowing any filling defects in the gelatine to be identified and distinguished from later bullet tracts.

v. Ballistic testing

**Fig. 3:**
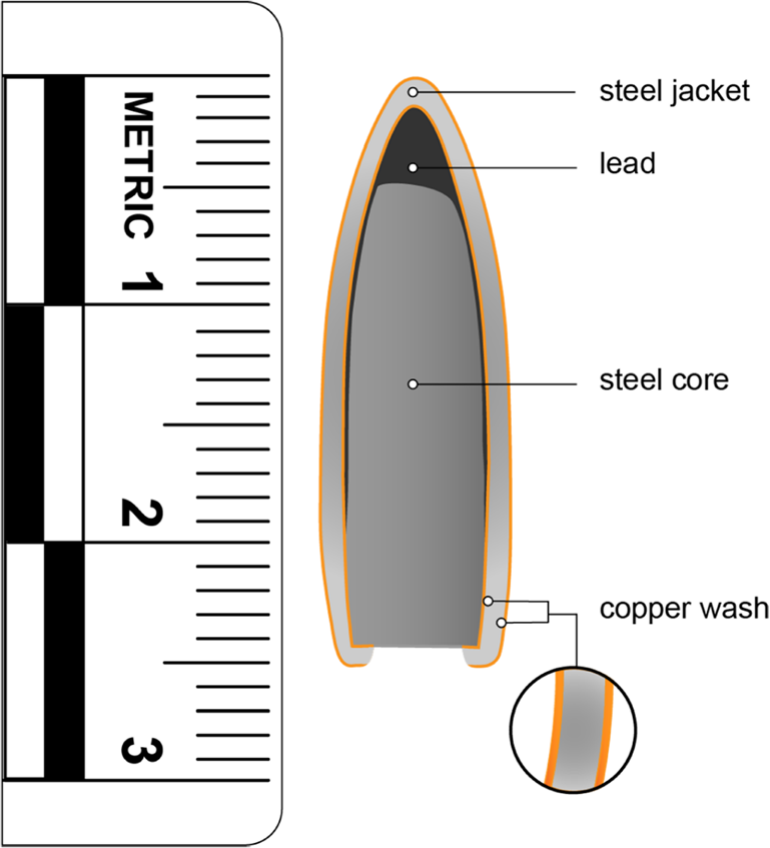
Details of the 7.62 x 39 mm Ukrainian MSC ammunition. (1) Composition confirmed using a Hitachi SU3500 scanning electron microscope with EDAX microanalysis system & TEAM software version 4.4. (2) Microhardness of core and jacket measured using an Indentec HWDM-7 apparatus with a diamond indenter (Indentec, Unit 30 Navigation Drive, Hurst Business Park, Brierley Hill, West Midlands, DY5 1UT UK). Mean hardness of core* is 207 HV, SD 18 HV and of jacket 199 HV, SD 9 HV. Lead hardness 3.9 HV. (*from *n* = 3 bullets using *n* = 3 measurement points from each core and *n* = 5 measurement points from each jacket)

The following day the models were shot at a range of 10 m from a No 3 Enfield proof mount fitted with an accurate barrel using 7.62 × 39 mm Ukrainian mild steel core (MSC) ammunition (Soviet State Factory, Lugansk, manufactured 1967)(mean impact velocity 652 m/s, SD 6 m/s; Fig. 3).This ammunition type was chosen as representative of those faced by UK armed forces and allies [36–39].

**Fig. 4:**
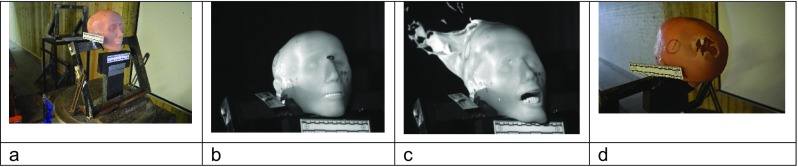
Face 1/Golf. **a** In situ at the range. **b** Image from V12 high-speed video immediately prior to bullet impact. **c** Impact event—bullet has exited the model; the temporary cavity develops within the gelatine and the skin is extended. **d** Resulting exit wound in model

Prior to each shot, the impact site on the model was confirmed using a sighting laser. The intended impact site was central into the frontal bone, below the post-mortem cut line, and around the level of the supraorbital margin. Projectile velocity was tracked using a Weibel Doppler, and impacts filmed using two Phantom high-speed cameras (from the front—V12, sample rate 28,000 frames per second, exposure 4 μs resolution 512 × 384; from the side—V1212, sample rate 37,000 frames per second, exposure 4 μs, resolution 512 × 384). Experimental setup and typical images from an impact event are shown in Fig. 4.

The condition of the models in situ post impact was recorded using a Nikon D3200 DSLR camera fitted with an AF-S NIKKOR 18–55-mm lens. The temperature of the gelatine was taken immediately post impact using a calibrated digital thermometer.

vi. Second set of CT scans

**Fig. 5:**
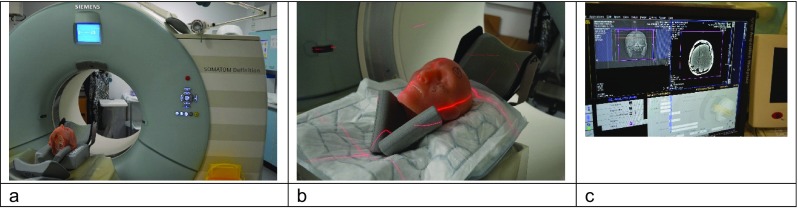
**a** Face 1/Golf in the CT scanner. (b) Detail of Face 5 /Kilo in the CT scanner. (c) CT work station displaying images from Face 2/Hotel; bullet tract and fractures are visible in the right-hand image on the screen

**Fig. 6:**
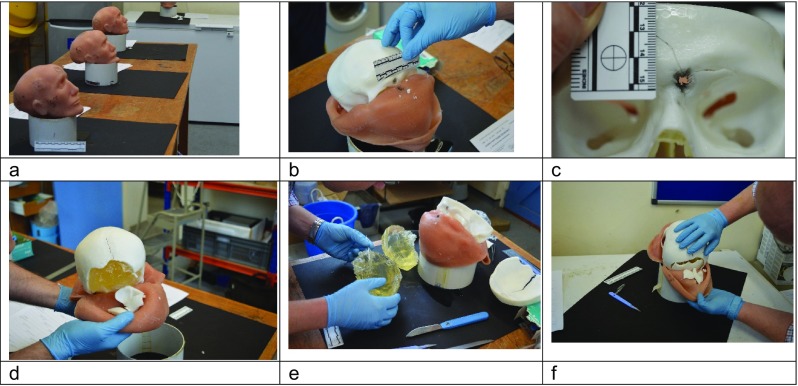
Pathologists’ examination of models after shooting. **a** Setup of examination room. **b** Entry wound in Face 3/India and underlying skull. **c** Detail of entry wound Face 6/Lima showing bullet wipe and radial fractures. **d** Corresponding exit wound, Face 6. **e** Examination of gelatine brain, Face 6. **f** Exit wound Face 2/Hotel

That evening the shot models were re-imaged at the Centre for Defence Imaging using the same CT protocols as for the first set of scans. Each model was scanned both without and with contrast material (Omnipaque™300 (Iohexol, GE Healthcare Inc)) injected into the wound tract (Fig. 5). A pilot project imaging a series of 20-ml syringes filled with Omnipaque™300 diluted with different amounts of 0.9% saline found a mixture of 40% contrast and 60% saline produced the clearest images with the least artefact. For this study, 20 ml of this mixture was gently introduced into wound tract using a syringe and a soft catheter, splitting 10 ml into the entry wound and 10 ml into the exit wound.

vii. The six models were then examined by two Home Office Forensic Pathologists with extensive experience of assessing ballistic injury. The pathologists were invited to conduct a formal ‘post-mortem’ examination of each model (Fig. 6) and score them using a 4-point Likert-type scale [40] (Table 1) similar to that used in our earlier work [4] but looking at more parameters (skin and soft tissue characteristics, entry wound, exit wound, fractures, wound tract, imaging).The score sheet also included space for comments if the pathologists wished to provide them. These are summarised in the ‘Results’ section below.

**Table 1:** Example Likert-type score sheet used by assessors. Parameters assessed were (i) skin and soft tissue characteristics, (2) entry wound, (3) exit wound, (4) fractures, (5) wound tract, and (6) imaging

**Figure 7.**
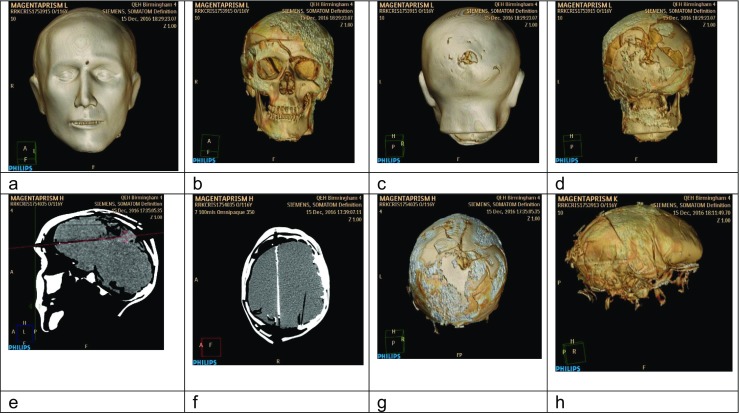
CT reconstruction images from Phillips Brilliance Extended Work Station; **a**–**d** show Face 6/Lima. **a** Entry wound. **b** Fracture patterns underlying entry site. **c** Exit wound. **d** Fracture patterns under exit site. **e**–**g** Face 2 /Hotel. **e** Sagittal view of bullet trajectory with fractures and exit wound. **f** Cross-sectional view of same features; OmnipaqueTM300 contrast present in the bullet tract. **g** 3D reconstruction of fractures. **h** Face 5/Kilo; 3D reconstruction of gelatine brain with posterior damage post shot.

Exit wound scoring chart—please tick one only.
1. This looks nothing like a real exit wound
2. This looks a bit like a real exit wound
3. This looks a lot like a real exit wound
4. This looks exactly like a real exit wound

viii. The pre- and post-shot CT scans were viewed by a Military Consultant Radiologist with extensive experience of ballistic injury imaging using OsiriXDICOM viewer (http://www.osirix-viewer.com, PixmeoSARL, 266 Rue de Bernex, CH-1233 Bernex, Switzerland). Tissue layers were removed from the images using Phillips Brilliance Extended Work Station (Koninklijke Phillips N.V., Amstelplein 2, 1096 BC Amsterdam, The Netherlands) and the underlying damage assessed as described by Myers et al. [41] and scored using the same sheets referenced above. Examples of the CT scans are shown in Fig. 7.

ix. The high-speed video images were reviewed to track the bullet trajectory through each of the models, assess if the impacts differed from one another and look for evidence of damage to the bullets.

## Results

**Fig. 8:**
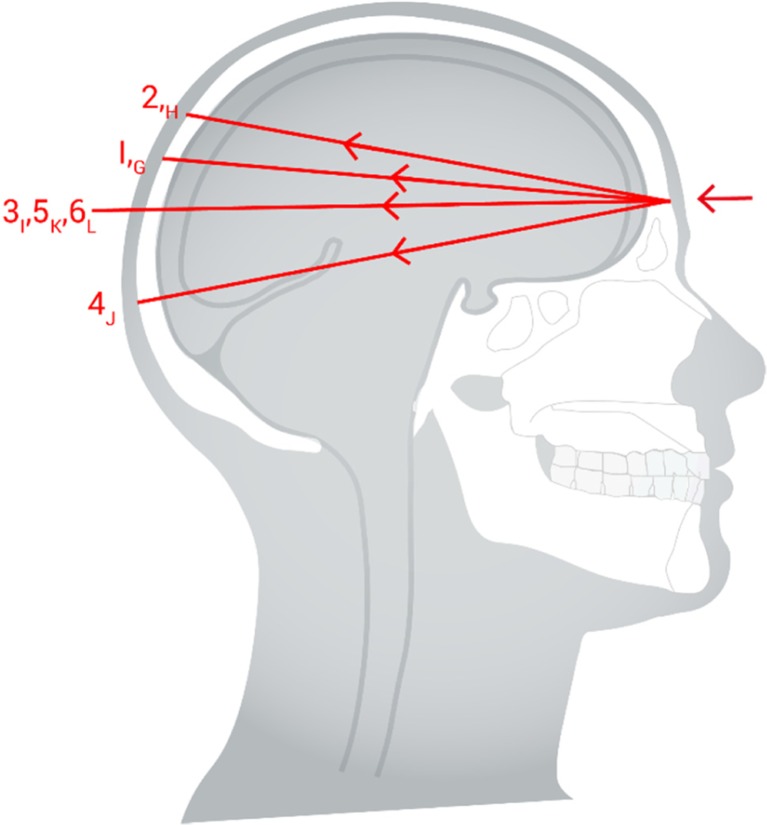
Summary of bullet trajectories from high-speed video images

Review of the high-speed video found that the bullets followed four slightly different trajectories. These are summarised in Fig. 8.

All the bullets with the exception of the one impacting Face 4 emerged intact from the models. Face 4 is considered further below under ‘Fracture Patterns’.

**Fig. 9:**
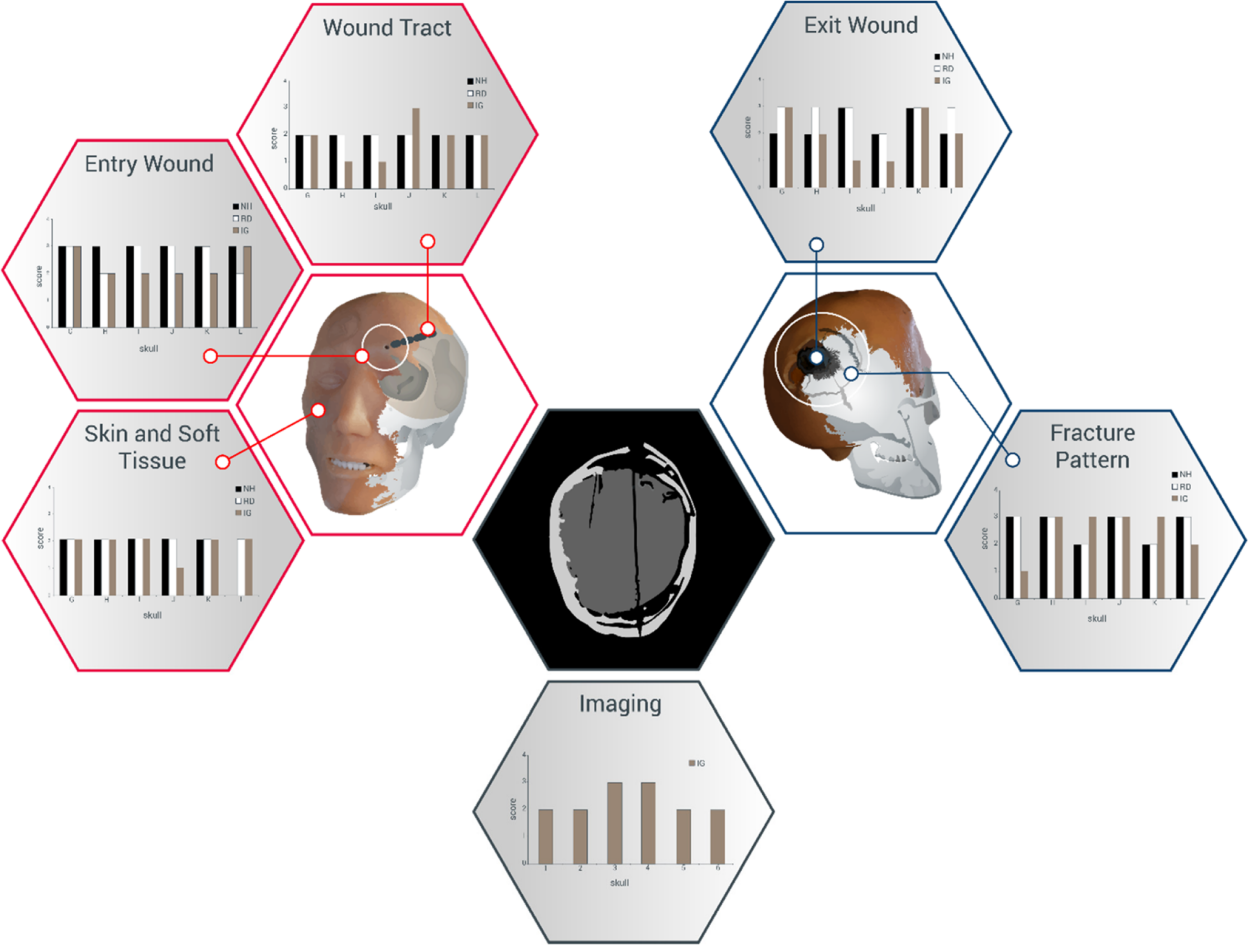
Results from Likert-type score sheets summarised as graphs. Assessors are designated by initials and colours (NH—N Hunt, RD—R Delaney, IG—I Gibb). Scores from NH and RD done from physical examination of models. Scores from IG done by examination of the CT scans

The scores from the Likert-type scales were collated in an Excel spreadsheet. These are summarised in Fig. 9.

The free text comments and notes made on the score sheets by the clinicians were also transcribed into an Excel spreadsheet so that comments about the wound characteristics and fracture patterns could be compared and assessed.

Each parameter (apart from imaging) could achieve a maximum score of 72 (i.e. 6 models, 3 assessors, maximum score of 4 from each assessor). The actual score obtained for each parameter was divided by 72 and multiplied by 100 to give an indication of how ‘real’ the combined assessors regarded each parameter to be (%). Imaging assessment was undertaken by only one assessor (IG) with a maximum possible score of 24.

No parameter scored the maximum 4 from any of the assessors.

With the small number of observations being considered, more complex statistical analysis was not appropriate.

### Skin/soft tissue appearance and feel: score 33/72 = 46%

The skin/soft tissue was mainly given a score of two by each assessor for each model, other than one which was not scored by one of the pathologists and another (Face 4) given a score of one based on the CT images by the radiologist. The radiology comment for Face 4 was the soft tissue over the frontal bones was too thick, which in turn exaggerated the wound tract through the soft tissue.

### Entry wounds: score 48/72 = 67%

Five of the tissue entry wounds (Faces 1 to 5) were described as ‘too small for 7.62 mm bullet’ by the pathologists. All were noted by the pathologists to have visible bullet wipe; Contusion and radial splits were present but required additional lighting and magnification to be seen well. On CT reconstruction, the bullet entry wound was described as ‘gaping open’ with the comment that real wounds often close down to a slit. The impact site on Face 6/Lima was more elliptical than expected and one of the pathologists (NH) felt this was due to its location over the medial aspect of the supraorbital ridge.

### Exit wounds: score 43/72 = 60%

Comments from all assessors were that the exit wounds in the soft tissue were generally more realistic than the entry wounds, although the overall score was lower. The larger wounds were regarded as more realistic (Faces 1, 2 and 5). Five wounds were noted to have everted margins and irregular edges, as is commonly seen in real wounds. Both Faces 3 and 4 were scored very low on CT examination with the comment that the exit wound in the soft tissue was not consistent with the underlying fractures, although Face 3 was described as realistic by the pathologists and scored well.

### Wound tracts: score 33/72 = 46%

Only one wound tract (Face 1) was described as realistic by one pathologist (RD). Two wound tracts (Faces 1 and 4) had fragments of bone and skin within the tract; features which are seen in real incidents. Bullets were noted to yaw at distances between 50 and 110 mm from entry into the gelatine. In five tracts, the damage to the distal end of the tract from the bullet yaw was such that detailed assessment was not possible by the pathologists (Faces 2, 3, 4, 5 and 6). From viewing the CT scans, IG noted that the spread of contrast within the gelatine was very different to real brain. The pathologists stated that where folds were present in the gelatine (due to the thin polyethylene bag in the skull, see ‘Methods’ section part iii above) tract assessment was impeded and that as the ballistic injury features in real brain are very different to those in gelatine [19], direct comparison was not possible.

### Fracture patterns: score 47/72 = 65%

**Fig. 10:**
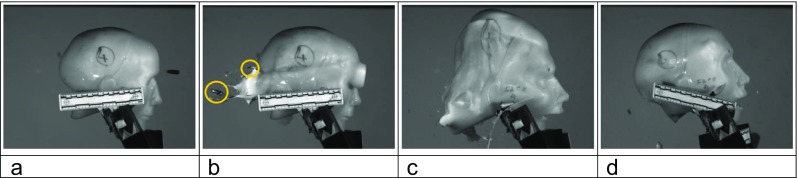
**a** Face 4, immediately before impact. **b** Bullet has exited damaged (left hand circle); bullet tip is visible separately (right hand circle); entry wound is still expanding. **c** Temporary cavity expansion. **d** Resting position after temporary cavity has collapsed down; skull fractures are visible through the synthetic skin and soft tissue

Five of the entry sites had associated radial fractures, although these were found more often by the pathologists than from the CT scans due to the soft tissue CT appearance being close to that of the synthetic bone as described above (Faces 1, 2, 3, 4 and 6). From the pathologists’ examinations, three of the entry sites (Faces 3, 5 and 6) had both internal and external beveling at the entry site. Two of the exit sites had external beveling (Faces 1 and 3), but for others, loss of material at the exit site made beveling assessment not possible. Three faces were described as ‘realistic’ by at least one pathologist (Faces 3, 4, 6). Face 4 was described as ‘realistic for the trajectory, including the palpable mid-face fracturing’. The pathologists noted the round had struck the right petrous ridge. On the high-speed video, this is the only round seen to have fractured on exit from the model (Fig. 10). As with previous work [4], the post-mortem cut present in the model impacted on some of the fracture propagation and was noted to be an issue in two of the models (Faces 5 and 6).

### Imaging: score 14/24 = 58%

While the CT images were able to produce good surface reconstructions, the skin/soft tissue properties were very close to those of the synthetic bone making reconstruction of the underlying structures difficult (e.g. Fig. 7). The key observation from the overall assessment of the CT reconstructions was that the exit fractures were most realistic while the skin wounds and wound tract were less so.

## Discussion

As far as possible, the models used in the current work were constructed to be identical but inevitably there were minor differences (such as with the creases in the gelatine fill). The bullet ‘strike’ point was consistent for each model but there was variation in the trajectories, fracture patterns and entry/exit wound appearances as shown by the assessments. Schyma et al. [17] also noted differences among their four models and suggested this might be due heterogenicity in the silicone coatings on the spheres.

Previous authors have suggested a high degree of authenticity for their ballistic injury models compared to actual wounds [3, 5, 7, 15]. Based on clinical experience in Afghanistan (2007–2014) of managing casualties soon after injury during battlefield evacuation, one of the authors of the current study (PFM) felt that the overall look and feel of our model after shooting (particularly the exit wound and fracture complex) was realistic.

None of the models scored a ‘4’ (‘exactly like a real injury’) in any of the parameters assessed, unlike our previous study which did not include soft tissue [4]. In our previous study [4], 23 of the 39 skulls assessed for fracture patterns were given a score of three by at least one of the five assessors and seven were given a score of four by at least one assessor. No skulls received the same scores from all five assessors. Figure 1 shows the ‘ideal’ characteristics of gunshot injuries to the head but as noted in the ‘Introduction’ to this paper, the literature demonstrates that there are exceptions reported to these appearances in both experimental work and actual cases.

The main critical comment from the pathologists was that the skin/soft tissue was ‘too stretchy’. The characteristics of the synthetic skin and soft tissue have major influences on the appearance of the entry and exit wounds. Rothschild [11, p257] states that the diameter of the central skin defect [Fig. 1, A] is generally smaller than that of the bullet as, after being extended, the skin recovers elastically following the bullet perforation. Even allowing for this, and the reported bullet hole variation in experimental cases [23], our entry wound defects are smaller than real injuries. (around 2 to 3 mm diameter skin defect). Previous work at the Impact and Armour Group has used food-grade swine tissue for ballistic experiments; mean entrance holes using similar ammunition were 4.8 × 5.1 mm (*n* = 3) [30]. The skin entry wounds do show many features of real gunshot wounds (including bullet wipe, Contusion and radial tears) but to a varying degree. The properties of the skin/soft tissue surrogate are suitable for clinical training (see ‘Methods’ section above) but at ballistic strain rates behave differently to real skin. The similarity of the skin to synthetic bone on CT imaging also made aspects of the CT assessment challenging.

A key function of the surrogate soft tissues was containing the majority of the fragments associated with the bone exit wound. Even with this, some of the material was lost making assessment of the exit characteristics difficult. In real cases, wound assessment can also be frustrated by lost fragments, surgical treatment and scavenger activity [6].

Smith et al. [24] compared ballistic impacts on polyurethane bone substitute [SYNBONE®] with those on cattle scapulae. Impacts on the synthetic bone with modern rifle bullets (7.62 × 51 NATO FMJ and .243 Winchester jacketed soft point) produced the expected bevelled margins. The bone surrogate used in the current work showed some of the elements of real bone injury although beveling at the entrance and exit sites was inconsistent, as can be the case in real ballistic events [13]. Overall, the reviewer response to the macroscopic fracture pattern in this work and the previous studies [3, 4] was positive, likely due to the anatomically correct features of the skull model used.

Gelatine 10% is a very different material to living brain and the wound tract produced in the model is not as complex as actual injuries. As noted above (imaging in ballistic investigations), real brain injury includes cortical contusions and bleeding from tissue shearing with associated oedema. Gelatine 10% does allow the formation of a temporary cavity and the production of realistic additional fractures as shown in Fig. 1 [4].

Our current study used a combination of physical assessments by pathologists and imaging assessments by a radiologist. Although Bollinger et al. [18] felt that imaging offered advantages over dissection, we agree with Karger [19] that the combination of methods is best as each can inform the other in searching out particular information (such as accurately locating intracranial fragments [21]).

A significant test for our model will be using it to recreate actual ballistic incidents and assessing how the CT images and injury patterns from the models compare with those from real cases, allowing ‘measurement and extrapolation’ as stated by Jussila [34].

## Conclusions

An anatomically correct synthetic skull with a surrogate skin/soft tissue layer was impacted with 7.62 x 39 mm bullets and the damaged assessed by two pathologists and a radiologist with experience of real gunshot wounds caused by similar ammunition. Drawing on two different clinical specialities has offered both contrasting and complementary views of the realism of the model.

The assessment was undertaken both by physical examination and CT imaging. The model showed some of the features of real wounds including entry and exit wound characteristics and macroscopic fracture patterns—but individual elements (including the size of bullet holes in the skin and synthetic bone beveling) need refinement. Testing the model against data from actual incidents will allow us to critically assess it further and undertake these refinements.

### Caveats

This paper only reports findings with one ammunition type fired at approximately 650 m/s. Other weapon systems or ammunition types may produce different results under these experimental conditions.
